# Optical Genome Mapping in Routine Human Genetic Diagnostics—Its Advantages and Limitations

**DOI:** 10.3390/genes12121958

**Published:** 2021-12-08

**Authors:** Paul Dremsek, Thomas Schwarz, Beatrix Weil, Alina Malashka, Franco Laccone, Jürgen Neesen

**Affiliations:** Institute of Medical Genetics, Center for Pathobiochemistry and Genetics, Medical University of Vienna, 1090 Vienna, Austria; thomas.schwarz@meduniwien.ac.at (T.S.); beatrix.weil@meduniwien.ac.at (B.W.); alina.malashka@meduniwien.ac.at (A.M.); franco.laccone@meduniwien.ac.at (F.L.); juergen.neesen@meduniwien.ac.at (J.N.)

**Keywords:** OGM, optical genome mapping, cytogenetics, cytogenomics, routine genetic testing, structural chromosomal aberrations, numerical chromosomal aberrations

## Abstract

In recent years, optical genome mapping (OGM) has developed into a highly promising method of detecting large-scale structural variants in human genomes. It is capable of detecting structural variants considered difficult to detect by other current methods. Hence, it promises to be feasible as a first-line diagnostic tool, permitting insight into a new realm of previously unknown variants. However, due to its novelty, little experience with OGM is available to infer best practices for its application or to clarify which features cannot be detected. In this study, we used the Saphyr system (Bionano Genomics, San Diego, CA, USA), to explore its capabilities in human genetic diagnostics. To this end, we tested 14 DNA samples to confirm a total of 14 different structural or numerical chromosomal variants originally detected by other means, namely, deletions, duplications, inversions, trisomies, and a translocation. Overall, 12 variants could be confirmed; one deletion and one inversion could not. The prerequisites for detection of similar variants were explored by reviewing the OGM data of 54 samples analyzed in our laboratory. Limitations, some owing to the novelty of the method and some inherent to it, were described. Finally, we tested the successful application of OGM in routine diagnostics and described some of the challenges that merit consideration when utilizing OGM as a diagnostic tool.

## 1. Introduction

Genomic structural variants (SVs) involve the loss, multiplication, rearrangement, or translocation of large genomic regions. SVs are common events and are in many cases associated with specific phenotypes and diseases [1]. Since the 1960s, karyotyping has been employed for clinical analyses to detect chromosomal numerical aberrations and SVs [2]. A karyogram is able to identify basic SVs involving deletions, duplications, translocations, and inversions of genetic material. However, depending on the banding technique used, the type of SV and the region in which it is located, the resolution of karyotyping is limited to approximately 3–10 Mbp [3]. In the 2000s, the more sophisticated array comparative genomic hybridization (aCGH) method was introduced as a new means for routine genetic diagnostics [4]. Based on DNA hybridization, aCGH offers a resolution of several hundreds to thousands of base pairs, depending on the number of probes used. This technique is powerful in regard to the identification of losses and gains of genetic material, but it is not able to detect balanced SVs, such as inversions and balanced translocations, or complex structural variants [3]. Currently, whole-genome sequencing (WGS) with second- (i.e., short-read sequencing) and third-generation (i.e., long-read sequencing) technologies are also capable of detecting SVs [5,6]. Although computationally challenging, this technology allows the detection of all types of SVs. Nevertheless, the relatively short DNA strands used for sequencing present a significant limitation; as the human genome consists of long regions of highly similar, often repetitive sequences, and these regions tend to be difficult or impossible to analyze using short molecules of DNA only [5]. As repetitive regions are often involved in the formation of SVs, their accurate mapping may contribute to the detection of SVs hitherto missed [7,8].

Optical genome mapping (OGM) is an approach to analyze large eukaryotic genomes and their structural features at a high resolution. It uses linearized strands of high molecular weight (HMW) DNA that are far longer than the DNA sequences analyzed in current second- and third-generation sequencing methods, achieving average read lengths in excess of 200 kbp. For comparison, the RS II system from Pacific Biosciences (Menlo Park, CA, USA) has an average read length of 10–16 kbp [9]. Oxford Nanopore Technologies (ONT, Oxford, UK) devices have a practical limitation of approximately 20 kbp if significant coverage of the human genome is required, although with considerable effort, a length N50 of 100 kbp is possible for low coverage of the whole human genome [10]. The usage of long molecules in OGM allows repetitive regions and other regions that are complicated to map to be spanned more easily than with short molecules. This leads to the creation of maps that may cover the whole arm of a chromosome and yet allow the detection of insertions and deletions as small as 500 bp [11]; other SVs need to be 30 kbp or larger to be detectable.

Recently, the system has been used to successfully detect the breakpoints of chromosomal translocations, for the diagnosis of facioscapulohumeral muscular dystrophy (FSHD) and has been suggested for use as a cytogenomic tool for prenatal diagnostics [12,13,14,15,16]. One study compared OGM using the Saphyr system to conventional point-of-care methods for the detection of 100 different chromosomal aberrations [17]. Hence, this technique may at least partially fill the gap between WGS and karyotyping for the detection of SVs.

This study aimed to assess the utility of the novel OGM technique in routine human genetic diagnostics. Although the idea of using OGM as a tool in routine diagnostics is not entirely new, we explore which types of SVs may be better analyzed by OGM than by existing techniques [12]. We evaluate how the Saphyr system fares compared to other methods used in routine diagnostics, its advantages and disadvantages, and whether it is able to replace or complement other techniques, with the aim of accelerating the turnaround time and improving the fidelity and clinical value of both prenatal and postnatal analyses.

## 2. Materials and Methods

### 2.1. Samples

This study includes OGM data from a total of 54 individuals, of whom 37 are patients at the Institute of Medical Genetics at the Center for Pathobiochemistry and Genetics of the Medical University of Vienna, and 17 are healthy probands and part of a control group.

### 2.2. DNA Extraction

Cell culture material and peripheral blood samples were either used immediately after recovery or frozen at −80 °C according to the manufacturer’s recommendations.

HMW DNA was extracted for OGM with the “Blood and Cell Culture DNA Isolation Kit” (Bionano Genomics, San Diego, CA, USA), which we later exchanged for the “SP Blood & Cell Culture DNA Isolation Kit” (Bionano), according to the manufacturer’s protocols (“SP Frozen Human Blood DNA Isolation”, “SP Frozen Cell Pellet DNA Isolation”, “SP Fresh Human Blood DNA Isolation Protocol”, “SP Fresh Cells DNA Isolation Protocol”, “Blood DNA Isolation Protocol”, or “Frozen Blood DNA Isolation Protocol”). The isolation kits differ greatly in execution time but showed otherwise no noticeable differences, especially regarding the quality of the resulting OGM data.

Briefly, the leukocytes in the blood samples were quantified by a HemoCue WBC Analyzer (HemoCue, Ängelholm, Sweden). Cultured cells were quantified using a hemocytometer. DNA from approximately 1.5 × 10^6^ cells was extracted by two different protocols: when using the “Blood and Cell Culture DNA Isolation Kit”, the cells were immobilized in agarose plugs and lysed by proteinase K; thereafter, the genomic DNA was washed, recovered, and quantified. When using the “SP Blood & Cell Culture DNA Isolation Kit”, the cells were lysed by proteinase K, and the genomic DNA was bound to a magnetic disk. Subsequently, the DNA was washed, recovered, and quantified.

In both protocols, the quantification of the extracted DNA was performed using the Qubit dsDNA BR assay kit on a Qubit 2.0 (Thermo Fisher Scientific, Waltham, MA, USA). The DNA extractions for all other molecular genetic test methods were performed by an automated DNA extractor according to the instrument protocol (chemagic MSM I, PerkinElmer, Waltham, MA, USA).

### 2.3. DNA Labeling and Further Processing for OGM

A total of 750 ng of genomic HMW DNA was labeled using the DLS DNA Labeling Kit (Bionano) according to the manufacturer’s instructions. Briefly, the DNA was labeled with a fluorophore by the methyltransferase DLE-1 at the recognition motif CTTAAG. This generates approximately 14–15 labels per 100 kbp when labeling human genomic DNA. Thereafter, the DNA was dialyzed, its backbone was stained, and finally the prepared DNA was applied to G1.2 flow cells (Bionano). The flow cell was then inserted into the Saphyr instrument, where the DNA was fed by electrophoresis into the nanochannels of the flow cell for linearization. A fluorescence microscope was then used to scan the DNA-filled nanochannels. The captured images were converted to electronic representations of the DNA molecules. The virtual DNA strands were then filtered and de novo assembled into maps.

### 2.4. Assembly of OGM-Data and Quality Metrics

The data acquired with the Saphyr instrument were processed with several software modules (Tools Version 1.6.1, Solve Version 3.6.1_11162020, RefAligner Version 11643, Pipeline Version 11646). Briefly, the raw data were filtered for a minimum length of 150 kbp and minimum of nine labels per molecule. The filtered molecules were de novo assembled, and the consensus maps of the molecules were aligned to the human reference genome GRCh38. Variants were detected by either of two software modules. The primary software module, called the “SV pipeline”, compares the maps to the aligned reference genome. There, patterns of markers from the maps deviating from the reference become apparent. A secondary software module, called the “CNV pipeline”, was also used. This module quantifies the mapped molecules and hence is able to detect gains and losses of several hundred kbp in size, similar to aCGH.

The results of the SV pipeline were then augmented by the “variant annotation pipeline”, which adds quality metrics for the called variants and supplies their estimated frequency in the human population based on an internal database. The optional step of filtering based on the frequency of the SVs in the internal database was not used. The SVs were detected by manual inspection of the generated data using the browser-based interface of the Bionano Access software (Version 1.6.1). Additionally, the system’s ability to automatically call the SVs was utilized. Automatic calling was based on the confidence scores and sizes of the SVs (insertions and deletions: confidence > 0, size > 500 bp; inversions: confidence > 0.7, size > 30 kbp; duplications: confidence = −1, size > 30 kbp; intrachromosomal translocations: confidence > 0.3; interchromosomal translocations: confidence > 0.65; CNV confidence > 0.99, size > 500 kbp). Additionally, each called SV was required to be spanned by > 5 strands of DNA.

The total amount of unfiltered DNA scanned by the system was 916 Gbp per sample on average. We aimed to achieve an “effective coverage of the reference” of >70× per sample. The effective coverage of the reference is defined as the total length of filtered (≥150 kbp) and aligned molecules divided by the length of the reference genome after de novo assembly [18].

### 2.5. Routine Diagnostic Methods

The routine diagnostic methods used to find or confirm the variants described in this study (i.e., aCGH, chromosomal analysis and fluorescence in situ hybridization (FISH), PCR and sequencing as well as multiplex ligation-dependent probe amplification (MLPA)) are described in the supplementary methods.

## 3. Results

### 3.1. Samples

A total of 54 samples were analyzed in this study. Routine human genetic diagnostics revealed a total of 14 SVs or chromosomal numerical aberrations of potential clinical relevance in 12 of the samples, which were chosen for further analysis. Two samples (S09 and S10) were analyzed without prior genetic testing (see Table 1). The samples were subjected to OGM to clarify questions regarding the detectability of the variants and whether the variants were called automatically and correctly by OGM, as well as to identify the additional information gained and the reliability of this information. The OGM data of the samples were evaluated with emphasis on the regions bearing the respective variants in a non-blinded fashion.

### 3.2. OGM Quality Metrics

The total molecule length N50, which is the point of half mass of the distribution of the molecules [18], was on average 195 kbp (ranging from 132 kbp to 393 kbp). The average label density of the raw molecules was 14.9 labels per 100 kbp (ranging from 12.6 to 16.2), leading to an average effective coverage of the reference genome of 110× (ranging from 70× to 193×) with an average diploid genome map length N50 of 63 Mbp (ranging from 40 Mbp to 81 Mbp) (*n* = 53). S01 was an outlier as its total molecule length N50 was only 95 kbp, resulting in an average effective coverage of the reference genome of 62× and an average diploid genome map length N50 of 12 Mbp. The average number of detected SVs after assembly and processing by the variant annotation pipeline was 5672 per analyzed sample (*n* = 54). The de novo assemblies of two samples took approximately 24 h. No filtering methods other than the recommended filtering by confidence score and size were applied.

### 3.3. Confirmation of Numerical and Structural Chromosomal Aberrations

It was examined whether several different types of variants could be detected and automatically called by OGM (see Table 1).

#### 3.3.1. Chromosomal Numerical Aberrations

**Figure 1:**
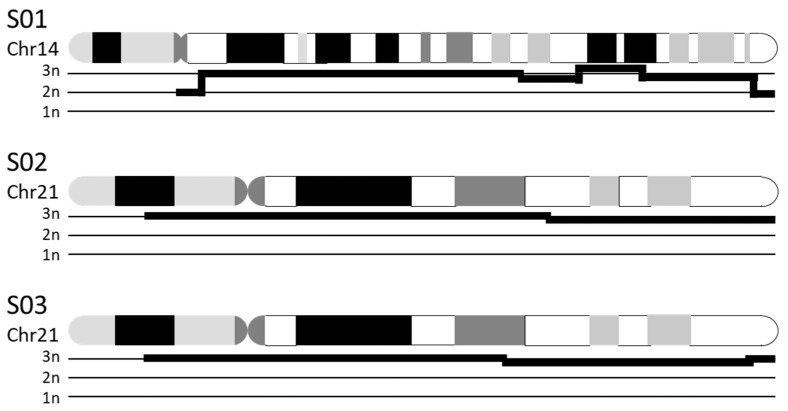
Schematic representation of Optical Genome Mapping (OGM) data to visualize trisomies. Free trisomies such as the depicted trisomies 14 (S01) and 21 (S02, S03) are detected by quantification of uniquely attributable genomic material, similar to the data obtained by aCGH. The graphs (bold black lines) underneath the ideograms (G-banding, black and gray: Giemsa positive, white: Giemsa negative) show the relative quantity of genetic material from chromosomes 14 and 21. In all three cases, the graphs show an elevated quantity of DNA, equaling three copies of each depicted chromosome. Centromeric regions and nonunique segments of the p-arms cannot be quantified, even though the continuous graphs suggest otherwise.

The ability to identify chromosomal numerical aberrations was tested for three samples with known trisomies (S01, S02, S03). In all cases, the Saphyr system was able to detect and call the gain of the euchromatic material that led to the trisomy. This was also the case for S01, even though the raw data acquired for this sample were of inferior quality (as discussed above). The software detects chromosomal numerical aberrations by its “CNV pipeline” module, which detects trisomies by their relative gain of uniquely attributable genetic material and provides data similar to that obtained from aCGH analysis (see Figure 1). As the genetic material of centromeric regions as well as other heterochromatic regions is not uniquely attributable, these segments of the chromosomes remain unquantified, and their gain can only be presumed. Additionally, to our understanding, as these structures cannot be covered by HMW DNA due to their size and repetitive nature, balanced translocations involving these structures (e.g., Robertsonian translocations) cannot be recognized by OGM. Hence, the trisomies of the analyzed samples were detected successfully but could not be recognized as free trisomies and were undiscernible from translocation-induced trisomies.

#### 3.3.2. Balanced SVs

**Figure 2:**
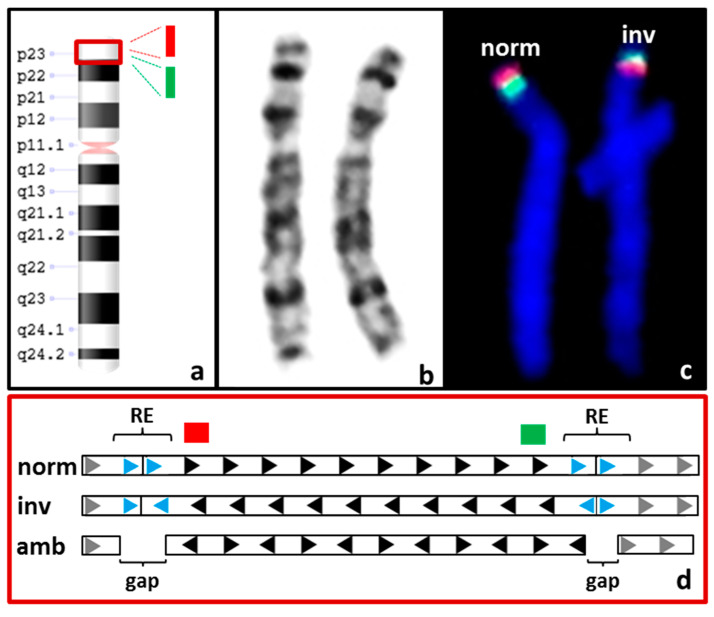
Inversion inv(8p23.1). The polymorphic inversion inv(8p23.1) of case S04 (**a**) is too small to be visible by karyotyping with GTG banding (**b**). Hence, it was initially detected by FISH analysis (**c**) using orange and green fluorescent probes that bind specifically to the inverted region (see orange and green fluorescent signals, blue: DAPI staining, the binding sites of the probes are indicated in (**a**,**d**) as green and orange blocks). Chromosome 8 carrying the inversion is depicted on the right (inv), with its green fluorescence signal above the orange signal, compared to normal chromosome 8 (norm) on the left side. (**d**) The repetitive elements (REs, represented by blue arrows) flank the segment that is proposed to be affected by the inversion (represented by black arrows). Gray arrows represent the surrounding sequences on chromosome 8p. When no inversion is present (norm), all arrows of the graphic representation are rightward oriented. When the inversion is present (inv), the arrows of the inverted segment are leftward oriented. The maps generated by OGM for case S04 did not span either RE (amb), leaving gaps and hence providing no frame of reference for the orientation of the segment between the REs. This creates ambiguity, where the inversion can be neither confirmed nor ruled out (represented by arrows with alternating orientation). By default, the Saphyr OGM system assumes the segment to be oriented correctly.

To examine the OGM system’s ability to detect balanced SVs, we used three samples bearing two different inversions and a reciprocal translocation (S04, S05, and S06). Case S04 bears a paracentric inversion within band p23.1 of chromosome 8. This is one of the largest polymorphic inversions known in humans, with an estimated frequency of 12–59% throughout different populations (see Figure 2) [20,21]. Although the inversion itself is considered benign, it may contribute to unequal recombination, thus causing deletions and duplications in the offspring of the heterozygous carrier of the inversion [22]. The inverted region is approximately 4.5 Mbp in size and is flanked on either side by low-copy large repeats (segmental duplications), which have estimated lengths of 1.3 Mbp (REPD, repeat distal) and 0.4 Mbp (REPP, repeat proximal) [20,23].

In sample S04, neither of these segmental duplications could be spanned by the OGM map due to their large size. Hence, the maps covering the short arm of chromosome 8 became discontinuous at these low-copy repeats. As no map spanned at least one of the segmental duplications flanking the inversion, it failed to be detected by OGM. To estimate the frequency at which these regions would not be spanned by OGM maps, we examined these regions in the other 53 samples included in this study. In 30 of 54 (55.6%) analyzed samples, neither of the segmental duplications could be spanned. In the remaining 24 samples, at least one repeat in at least one allele was spanned. Visual inspection of the OGM data indicated that six samples showed evidence for polymorphic inversion at 8p23.1, but the SV pipeline called this inversion in only one instance.

It is conceivable that samples with an above-median molecule length or effective reference coverage would be more likely to successfully span the segmental duplications. Based on the molecule lengths and coverages we achieved, we found that the repetitive elements were more often spanned in samples with a higher coverage; however, such a relationship was not observed for molecule length (see supplementary Figure S1).

In sample S04, the maps covering the short arm of chromosome 8 are otherwise continuous. Hence, it could be inferred that the inversion detected by FISH analysis has breakpoints within the segmental duplications. Confirmation of the location of the breakpoints by PCR was not possible due to the large sizes of the segmental duplications in which the breakpoints are believed to be located.

**Figure 3:**
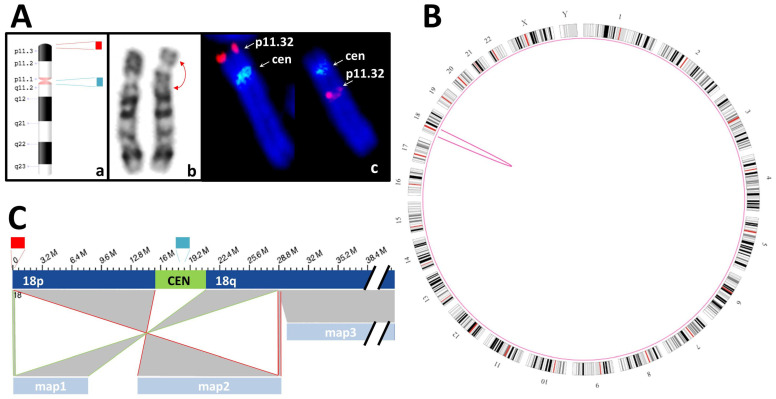
Inversion inv(18)(p11.3q12). (**A**): The pericentric inversion inv(18)(p11.3q12) of case S05 (a) can be detected by karyotyping with GTG banding (b) as well as by FISH analysis (c). The latter was done using orange and green fluorescent probes that bind specifically to the subtelomeric region of the p-arm of chromosome 18 and the centromere, respectively (see orange and green fluorescence signals, blue: DAPI staining). Normal chromosome 18 is on the left, whereas chromosome 18 carrying the inversion is on the right. (**B**): Circos plot with the ideograms of the 24 chromosomes (G-banding, black and gray: Giemsa positive, white: Giemsa negative, red: Centromere), generated by the Saphyr system, showing a pink arc at chromosome 18 protruding inwards from the breakpoints of the inversion. (**C**): Representation of a portion of chromosome 18 and the corresponding OGM maps (18p: full p-arm of chromosome 18; CEN: centromere; 18q: q-arm of chromosome 18, with its distal segment cropped; map1 through map3). The maps are connected to the segments with which they align by gray areas. Map1 aligns to a small, distal segment of the p-arm as well as to a large portion of the proximal q-arm (highlighted by green lines). Map2 aligns to a small segment of the q-arm as well as to a large portion of the proximal p-arm (highlighted by red lines). Map3 covers the rest of the q-arm. Maps 1 and 2 suggest that a pericentric inversion has taken place. As they are not continuous but separated by the centromere, the OGM system calls the SV as two intrachromosomal translocations.

The pericentric inversion on chromosome 18 of case S05 could be detected and called by OGM, and its breakpoints could be specified. However, no single map covers the entire inversion, as it incorporates the centromere, which cannot be spanned by the maps generated by the OGM system used here (see Figure 3). This causes the SV pipeline to fail to identify it as inversion and instead call it as two intrachromosomal translocations. Nonetheless, the SV can be identified as an inversion, especially in combination with karyogram analysis. To confirm the positions of the breakpoints indicated by the OGM data, specific PCR primers were designed to amplify the regions containing the breakpoints. To achieve reliable amplification, we designed the primers to bind at a considerable distance from the indicated coordinates of the breakpoint to account for possible inaccuracies in the coordinates. We deemed it ideal for the primers to flank the leftmost and rightmost intact markers. In doing so, we acquired amplicons that had lengths of 7.4 kbp (p-arm) and 3.4 kbp (q-arm) and confirmed the validity of the Saphyr system for identifying breakpoints. Sequencing of the PCR products led to the exact definition of the inverted material and its position at chr18:190,139→28,755,409 with 54 bp of unmappable DNA between the breakpoints and chr18: 28,755,492→190,143 with a gap of 44 bp of unmappable DNA.

**Figure 4:**
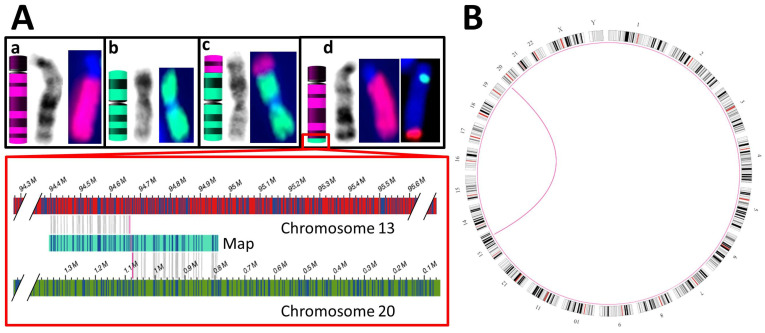
Translocation t(13;20)(q32;p13). (**A**): Normal chromosomes 13 (a, pink) and 20 (b, green) are depicted from left to right as ideograms, stained with GTG banding and labeled with whole-chromosome paint (WCP) FISH probes specific for chromosomes 13 and 20, respectively. In (c), a variant chromosome 20 is depicted, carrying at its p-arm the terminal portion of the q-arm of chromosome 13. This portion is detectable by GTG banding and WCP-FISH. In (d), the variant chromosome 13 is depicted, carrying at its q-arm the terminal portion of the p-arm of chromosome 20. This portion is too small to be detectable by either GTG banding or WCP-FISH. Therefore, FISH probes specific for the subtelomeric region of the p-arm of chromosome 20 (red) and the centromere of chromosome 13 (green) were used. In the rightmost image of (d), it is shown that chromosome 13, labeled green at its centromere, carries the subtelomeric region of the p-arm of chromosome 20. The red box shows a close-up view of this breakpoint: here, the left portion of an OGM map (light blue) is aligned to the q-arm of chromosome 13, whereas its right portion is aligned to the p-arm of chromosome 20 (alignment matches indicated by vertical gray lines). The transition between alignments to chromosome 13 and chromosome 20 delineates the breakpoint (shown as vertical red lines). Notably, the map covering the breakpoint is only 500 kbp in length and does not extend far beyond the breakpoint. This is the normal behavior of the employed assembly algorithm. The remaining chromosome is covered by other maps not shown in this image. (**B**): Circos plot generated by the Saphyr system, showing a pink arc between chromosomes 13 and 20, protruding inwards from the breakpoints of the reciprocal balanced translocation.

The balanced translocation of chromosomes 13 and 20 (S06) was initially detected by karyotyping in combination with FISH analysis and confirmed by OGM (see Figure 4). The position of the breakpoints identified by OGM was indirectly confirmed by aCGH analysis of a relative of S06. She was shown to carry both a duplication of a terminal portion of chromosome 13 and a deletion of a terminal portion of chromosome 20, corresponding to the duplication and the deletion to be expected from the unbalanced segregation of the translocation (Table 1).

The pericentric inversion and translocation were shown to not disrupt any genes that are currently considered clinically relevant. Hence, they can both be classified as likely benign with a high degree of certainty. Nevertheless, these variants remain associated with a risk of birth defects due to unbalanced segregation or unequal recombination in the offspring of their carriers.

#### 3.3.3. Large Unbalanced SVs

SVs routinely detected by aCGH can also be detected by OGM, whereas OGM offers the additional benefit of localizing gained material. To review the ability of the OGM system to detect large unbalanced SVs, four samples bearing a total of 1 deletion and 4 duplications > 90 kbp in size were analyzed (S07, S08, S10, S11). The 5 SVs of S07, S08, and S11 were all initially detected by aCGH. The duplications were all successfully confirmed by OGM. However, the deletion of case S11 could not be confirmed, as it was located in the subtelomeric region of the p-arm of the X chromosome, which was not covered by any map for this sample. This was not a rare event among the samples analyzed here; these regions (specifically, the region chrX:352,000–446,000 and the *CSF2R* gene) were not covered in 43 of 54 cases analyzed (79.6%, see supplementary Figure S1). We found that the 11 samples with coverage of this region had a slightly elevated coverage of the reference when compared to the noncovered samples, but elevated molecule length was not associated with coverage of the region (see supplementary Figure S1).

Subjects S09 and S10, the parents of S08, were analyzed by OGM only. Subject S10 was found to carry both duplications that were found in S08, whereas S09 was found to carry neither. Hence, S08 was shown to have inherited both duplications from S10.

Upon manual inspection of the OGM data, all duplications were confirmed, and the additional material was shown to be located in tandem with the original sequence. However, in several instances, the SV pipeline failed to call the duplications correctly. In cases S08 and S10, a duplication of 6.1 Mbp of the X chromosome was incorrectly called as an intrachromosomal translocation. In case S07, a 7.3 Mbp-sized duplication was not called by the SV pipeline at all, as was the case with a 16.1 Mbp duplication of case S11, both located on the X chromosome. In all cases, the duplications were called by the CNV pipeline, as their sizes were well above its threshold of 500 kbp.

All of the duplications could be confirmed, and the obtained genetic material was found to be inserted in tandem. Hence, at the resolution achieved by the Saphyr system, no gene appeared to be disrupted. One duplication of a portion of the X chromosome (S08, S10) involved the proximal segment of the *ATRX* gene. The disruption of this gene is considered clinically relevant, leading to an X-linked mental retardation disorder [24]. However, OGM showed that the duplicated material was not inserted within *ATRX*, suggesting that the gene remained intact and unaffected by the duplication.

#### 3.3.4. Small Unbalanced SVs

**Figure 5:**
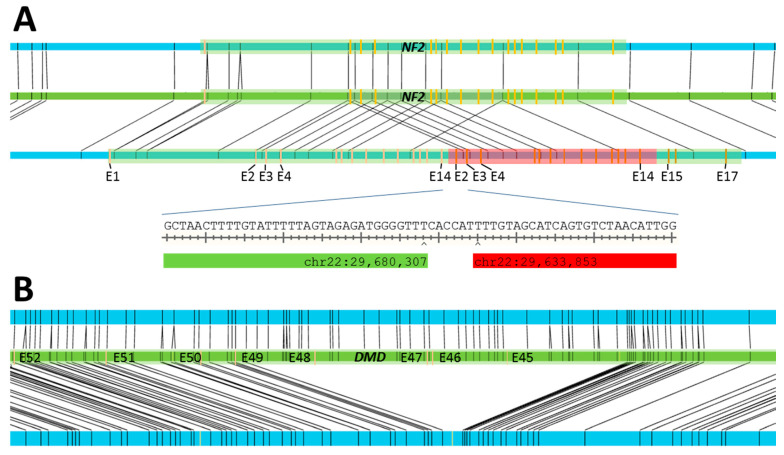
Schematic representation of small-scale SVs. (**A**): The green tracks show the reference genome ChGR38 at the position of the *NF2* gene of S12, with the gene itself shown as green widening of the track. Its exons are marked in orange. The OGM labels are marked in black. The blue tracks above and below the reference track show the two OGM maps covering the region and representing the two alleles present. They are likewise marked with the exons of *NF2* and the OGM labels. OGM labels aligned between a map and the reference track are depicted as black lines connecting them. The map above the reference track shows the normal allele, whereas the map below contains a tandem duplication. The duplicated portion of the reference is indicated by the alignment of each of its labels with two labels of the map. The duplicated portion of the *NF2* gene is situated between exons 14 and 15, thereby disrupting the gene (highlighted in red). The exact location of the breakpoint between exons 14 and 2, as determined by sequencing, is depicted below. (**B**): The deletion of several exons of the *DMD* gene of S13, shown using the same scheme as in (**A**).

To review the ability of the OGM system to detect small unbalanced SVs, three samples bearing deletions and duplications, all of which were initially detected by MLPA analysis, were analyzed (S12, S13, S14). They comprised a partial duplication affecting exons 2–14 of *NF2*, leading to the disruption of the gene and causing its carrier to develop neurofibromatosis type II (S12, see Figure 5A); a partial deletion affecting exons 45–48 of *DMD*, increasing the likelihood that the female carrier might have male offspring suffering from Duchenne muscular dystrophy (S13, see Figure 5B); and a deletion affecting *TNXA*, *TNXB*, and *CYP21A2* within the RCCX module of the MHC class III region (S14). This latter deletion is similar to others in this highly variable and repetitive region that are known to cause the formation of the chimeric gene *TNXA/TNXB* [25], which may lead to congenital adrenal hyperplasia in combination with connective tissue dysplasia.

All SVs could be confirmed successfully by OGM. However, the deletion affecting *TNXA*, *TNXB*, and *CYP21A2* was indicated to be present in 26.3% of the control samples provided in Bionano’s internal database. Hence, if this database had been used to filter out common SVs with a frequency of more than 26% (or a lower threshold), this deletion would have been missed. This could mean that the clinically relevant deletion present in S14 cannot be distinguished from benign polymorphisms at adjacent locations by the Saphyr system, making the system unsuitable for its detection.

To confirm and further characterize the positions of the breakpoints of the SVs given by OGM, PCR primers specific for the regions containing the breakpoints were designed. Primers flanking the leftmost and rightmost intact OGM markers were chosen, and the amplicons were expected to have lengths of approximately 12 kbp (S12), 10 kbp (S13), and 7.5 kbp (S14). To reduce the size of the amplicon of S12, the results of the MLPA analysis were incorporated into the primer design, leading to the design of primers with an expected amplicon size of 2.7 kbp. Amplicon sequencing confirmed the results of the OGM data for cases S12 and S13 and further specified the breakpoints of their SVs (S12, duplication affecting *NF2*: chr22:29,680,307–29,633,853; S13, deletion affecting *DMD*: chrX:31,845,259–32,019,560). Despite our attempts, we were unable to obtain and map an amplicon spanning the deletion affecting *CYP21A2*. We believe that the highly repetitive sequences found in this region prevented its analysis.

## 4. Discussion

This study aimed to evaluate the use of OGM in routine human genetic diagnostics to meaningfully complement or even replace already established diagnostic methods.

Karyotyping is one of the most basic first-line prenatal and postnatal diagnostic methods and is capable of detecting most types of balanced and unbalanced SVs as well as numerical chromosomal aberrations in clinical genetics. Its prime limitation is its coarse resolution of 3–10 Mbp or even lower when prenatal material is analyzed [3,26]. To overcome this poor resolution, karyotyping may be combined with aCGH, which greatly improves the rate of detection of unbalanced SVs. However, this approach does not improve the poor resolution of balanced SVs. In stark contrast to karyotyping, the Saphyr system has extremely high resolution and is able to detect balanced and unbalanced SVs as small as 30 kbp, making it superior to karyotyping in this regard [11]. Despite the significant advantage in resolution, OGM does have inherent limitations relative to the core strength of karyotyping in the detection of aneuploidies. As shown in this study, OGM can detect numerical chromosomal aberrations only by its CNV pipeline, which uses quantification of uniquely attributable genetic material to detect variants, similar to aCGH and most noninvasive prenatal testing (NIPT) approaches [27]. Hence, triploidies and higher-order polyploidies are undetectable with the current system [12]. This limits the scope of OGM when applied prenatally, as triploidies are of diagnostic relevance and can be found in 2–3% of pregnancies and in approximately 8% of miscarriages [28,29,30]. Additionally, as for aCGH, we expect difficulties in detecting low-level mosaicism of variants that are solely detected by the CNV pipeline, as seen in earlier data published by [31]. This would include, besides aneuploidies, unbalanced SVs that were missed by the SV pipeline, such as the duplications of cases S07 and S11. In contrast, SVs that are detected by a change in the OGM pattern have been shown to be detectable in low-level mosaics at frequencies of >5% [31,32]. The detection of mosaic chromosomal aneuploidies is especially important when analyzing chorionic villi, as placental mosaicism may occur in approximately 1–2% of samples [33,34]. It may also be clinically relevant in postnatal cases, although its prevalence is currently believed to be low [35,36]. Other variants that appear to be undetectable by OGM are Robertsonian translocations (RTs) and other whole-arm translocations that involve the centromere, which cannot be covered by the OGM maps we achieved with the Saphyr system. An estimated 0.1% of the general population are carriers of RTs, which makes RTs a fairly common type of SV [37]. RT carriers can have fertility challenges and an increased risk of trisomy in their offspring; therefore, the detection of RTs is critical [38]. Additionally, identifying a patient’s trisomy as stemming from familial RT has a significant impact on the recommendation of further testing to avoid reoccurrences [38].

Another constraint of karyotyping is the need for viable sample tissue for cultivation and the associated time needed to cultivate the material to obtain metaphases for analysis, which is approximately 72 h for lymphocytes [39] and significantly longer for prenatal materials. In our experience, cultivation of amniotic cells requires 8–14 days, and cultivation of chorionic villi requires 2–3 weeks. To alleviate the resulting long turnaround times for prenatal karyotype results, genetic laboratories may opt to complement karyotyping with other methods to supply the patient with more immediate, albeit preliminary, results. Among the most prevalent methods are interphase FISH analysis and quantitative PCR, which both may detect common aneuploidies, as well as the preparation of metaphases directly from chorionic villi [40,41,42]. All of these techniques may provide a preliminary result within 24 h. In contrast to karyotyping, which requires a long cultivation time to produce materials for analysis, OGM does not require any cultivation before sample processing, and according to our experience, de novo assembled genome data from OGM may be available in 4–6 days (the time required for the isolation and labeling of the HMW DNA and its subsequent analysis on the flow cell, as well as the rendering of the de novo assembly). This makes it considerably faster than karyotyping of prenatal samples, where speed is already a relevant factor. However, in our experience, the quantity of sampled prenatal material is often not sufficient for both isolation of HMW DNA (1.5 million amniotic cells or 10 mg chorionic villi, according to the Bionano protocols) and cultivation of cells for karyotyping [43]. As discussed above, OGM may not be suitable to fully replace karyotyping, depending on the indication. In these cases, cultivation of sampled material may be required to generate sufficient HMW DNA, which eliminates the time advantage of OGM over karyotyping. In addition, the Saphyr instrument used in this study requires approximately 24 h to gather sufficient genomic data for de novo assemblies of two samples; therefore, its throughput may be a limiting factor. Currently, Bionano is offering a new generation of Saphyr instruments that can hold up to six samples, which may be helpful in small-scale laboratories. However, these instruments can still analyze only one sample at any given time.

Improved coverage of large, repetitive structures such as segmental duplications is one of the prime advantages of the HMW DNA used in OGM. Coverage of segmental duplications is of genetic and clinical importance, as these regions may be involved in the formation of SVs and harbor SV breakpoints [8]. Nevertheless, even with the superior read lengths provided by OGM, we were not able to span every segmental duplication in all instances. In this study, the REPD and REPP segmental duplications of region p23.1 of chromosome 8 could not be covered in the majority of the samples analyzed (see supplementary Figure S1). Therefore, the large polymorphic inversion flanked by these segmental duplications was undetectable in these samples, as was shown in case S04 [20,21]. As this inversion is believed to cause unbalanced SVs in the offspring of its carriers, verifying its presence may be an objective in a routine genetic laboratory; often, this can be achieved by FISH analysis [22]. Overall, we do not deem the possibility that a segmental duplication might mask a balanced SV to be a significant drawback. However, when large balanced SVs are the target of the analysis (e.g., in recurrent pregnancy loss), this possibility should be accounted for, and other methods, such as karyotyping or FISH, should be considered.

OGM is comparable to aCGH in terms of their resolution for detecting SVs. The advantage of OGM over aCGH is the capability to detect balanced SVs, localize additional material, and potentially localize breakpoints at a high resolution. This is a significant benefit in clinical diagnostics, as balanced SVs have been shown to contribute significantly to congenital anomalies by disrupting genes and long-range regulatory interactions [44,45]. In the case of S05, we classified the pericentric inversion of chromosome 18 as likely benign, as the OGM data could rule out the disruption of known regions of clinical relevance by either breakpoint. This was also true for the balanced reciprocal translocation of chromosomes 13 and 20 found in case S06.

Although gains of genetic material may be detected by aCGH and OGM alike, aCGH cannot localize the additional material. A conclusive demonstration that the insertion of the additional material disrupts or avoids relevant genes and regulatory elements may help with the assessment of the clinical relevance of the duplication. In case S12, it was shown by OGM that the partial duplication of *NF2* was the cause of the neurofibromatosis of the patient, as the gene was disrupted by the insertion of the duplicated material. However, in case S08/S10, a duplication partially affecting *ATRX* was found to not disrupt the gene, which reduces the likelihood of the duplication being pathogenic. Hence, the ability of OGM to localize gained sequences can be considered a significant benefit.

In summary, the use of OGM to confirm and clarify SVs detected by aCGH seems worthwhile. However, in our opinion, its use as a first-line technique in lieu of aCGH would require significant effort; more experience with the artefacts associated with this technique is needed. Poor coverage of regions such as the subtelomeric region of the X chromosome, which led to a failure to detect the deletion in case S11, should be compensated for by different methods if the need arises. A similar approach should be used when frequent, benign SVs may pose a risk of masking similar pathogenic SVs. We observed this in case S14, where a deletion led to the formation of the chimeric gene *TNXA/TNXB*.

Additionally, there is the need for a system to filter the approximately 5500 SVs per sample, such as filtering by a database of samples representing the general population. However, to date, there is no publicly available database of balanced SVs (> 50 kbp in size) [6]. The database included in the Saphyr system currently comprises only a limited number of samples (analyzed with different techniques and enzymes, totaling approximately 300 samples) [12]. This relatively small number of samples may lead to skewed frequencies of SVs that are not representative of their true occurrence in the general population. Additionally, this database contains only the frequencies of the called variants and does not allow the inspection of the database entries. Hence, for a reliable evaluation of OGM data, we deem it necessary to develop and establish a curated database. To increase the power of such data collection, it may be helpful to share it within the community.

## 5. Conclusions

Here, OGM with the Bionano Saphyr system has proven to be a valuable tool to confirm the SVs initially detected by other diagnostic means. In almost all cases discussed here, confirmation by OGM came with the additional benefit of a significant gain of valuable genetic information. This benefit centers on the acquisition of high-resolution breakpoints of the SVs detected, which often allow the determination of whether genes or regulatory elements are affected and whether complex rearrangements are present. Furthermore, the identification of breakpoints is often precise enough to allow confirmation by further specific applications of additional techniques, such as PCR, as was successfully done here in cases S05, S12, and S13. This allows an even more detailed analysis by sequencing as well as the establishment of specific diagnostic PCR tests, e.g., preimplantation analysis, as was achieved for S12.

In the cases described, the SVs of interest were all known in advance, which allowed us to focus our analyses accordingly. However, when this is not the case, a more comprehensive interpretation of the OGM data becomes necessary. The SV calling methods that are integrated into the Saphyr system seem to be somewhat incomplete. In cases S07, S08, S10, and S11, large duplications could not be reliably called by the SV pipeline. In several instances, an inversion in region p23.1 of chromosome 8 was not called, although it was visible upon manual inspection of the OGM data. In case S11, a deletion could not be detected because the corresponding region was insufficiently covered. Hence, unless this OGM system is used to analyze a confined region or for a specific task, such as the diagnosis of FSHD, we propose that it should be used in conjunction with more traditional diagnostic methods [13]. We believe that this OGM system may be successfully combined with other techniques, complementing karyotyping and aCGH, depending on the objective. Another possible application of OGM would be to combine its data with sequencing data generated by Whole Exome Sequencing or WGS [46].

Finally, we showed the high value of this OGM platform, which we believe to be a useful supplement to the existing types of clinical genetic methods available. Currently, for routine human genetic diagnostics, we consider this highly promising novel technology can best express its full potential as part of a sound array of other diagnostic techniques. The widespread introduction of OGM as a new diagnostic tool will depend on the speed of the accumulation of further experience and the understanding of its benefits and capabilities. Furthermore, it is reasonable to expect improvements in the bioinformatic tools available for the analysis of the collected data.

## Figures and Tables

**Table 1:** List of the numerical and structural variants found by routine diagnostic means to be confirmed by OGM.

ID	Results of Routine Methods	Results of OGM
S01	K ^1^: 47,XX,+14	CNV ^2^: duplication of chr14:19,922,034–104,122,329 (i.e., majority of chr14)
S02	K: 47,XX,+21	CNV: duplication of chr21:13,033,053–46,697,230 (i.e., majority of chr21)
S03	K: 47,XY,+21	CNV: duplication of chr21:12,406,577–45,259,300 (i.e., majority of chr21)
S04	F ^3^: 46,XX.ish inv(8)(p23.1)(p23.1)(RP11-399J23+)(p23.1)(RP11-589N15+)	CNV + SV ^4^: not called and not detectable upon manual inspection
S05	K + F: 46,XY,inv(18)(p11.3q12?).ish inv(18)(p11q11)(D18Z1+)(18)(p11.3)(D18S1244+)	SV: intrachromosomal translocations from chr18:191,456 to chr18:28,753,623 and from chr18:192,099 to chr18:28,753,623
S06	K + F: 46,XX,t(13;20)(q32;p13) & aCGH ^5^ of a relative: duplication of chr13:94,677,014–114,327,680 and deletion of chr20:80,100–1,076,209	SV: interchromosomal translocations from chr13:94,664,378 to chr20:1,076,206 and vice versa
S07	aCGH: duplication of chrX:89,800,893–97,156,872	CNV: duplication of chrX:89,708,306–97,160,715 SV: not called but detectable upon manual inspection
S08	aCGH: duplication of chr14:188,966,428–190,415,619 and chrX:71,670,725–77,748,054	CNV + SV: duplication of chr4:188,157,784–189,603,002 and chrX:71,651,551–77,757,633
S09	n/a, father of S08	unremarkable
S10	n/a, mother of S08	CNV + SV: duplication of chr4:188,157,784–189,603,002 and chrX:71,651,551–77,757,633
S11	aCGH: deletion of chrX:352,452–446,323 and duplication of chrX:124,255,330–140,379,126	deleted region is not covered by OGM maps CNV: duplication of chrX:124,262,540–140,175,735 SV: duplication not called but detectable upon manual inspection
S12	MLPA ^6^: duplication of exons 2–14 of *NF2*	SV: 34.6 kbp duplication at chr22:29,636,202–29,670,823
S13	MLPA: deletion of exons 45–48 of *DMD*	SV: 174 kbp deletion at chrX:31,841,660–32,025,691
S14	MLPA: deletion of at least exons 3–7 of *CYP21A2* and deletion of at least exons 35–45 of *TNXB*	SV: 32.8 kbp deletion at chr6:32,012,952–32,045,806, found in 26.3% of samples in database

^1^ K: detected by karyotyping, nomenclature according to ISCN [19]; ^2^ CNV: CNV pipeline; ^3^ F: detected by fluorescence in situ hybridization; ^4^ SV: SV pipeline; ^5^ aCGH: detected by array comparative genomic hybridization; ^6^ MLPA: detected by multiplex ligation-dependent probe amplification. Reference genome for all genomic positions: GRCh38.

## Data Availability

The data presented in this study are available on request from the corresponding author.

## Supplementary Materials

The following are available online at https://www.mdpi.com/article/10.3390/genes12121958/s1, Figure S1: Boxplots illustrating two regions that were difficult to cover; Supplementary Methods.
