# Supplementary material for: Optical Genome Mapping in Routine Human Genetic Diagnostics—Its Advantages and Limitations
Source: Genes (Basel). 2021 Dec 8;12(12):1958. doi: 10.3390/genes12121958 (PMC8701374; doi:10.3390/genes12121958)
Supplement: Supplementary file 1 [file genes-12-01958-s001.zip › genes-1489756-supplementary.pdf]

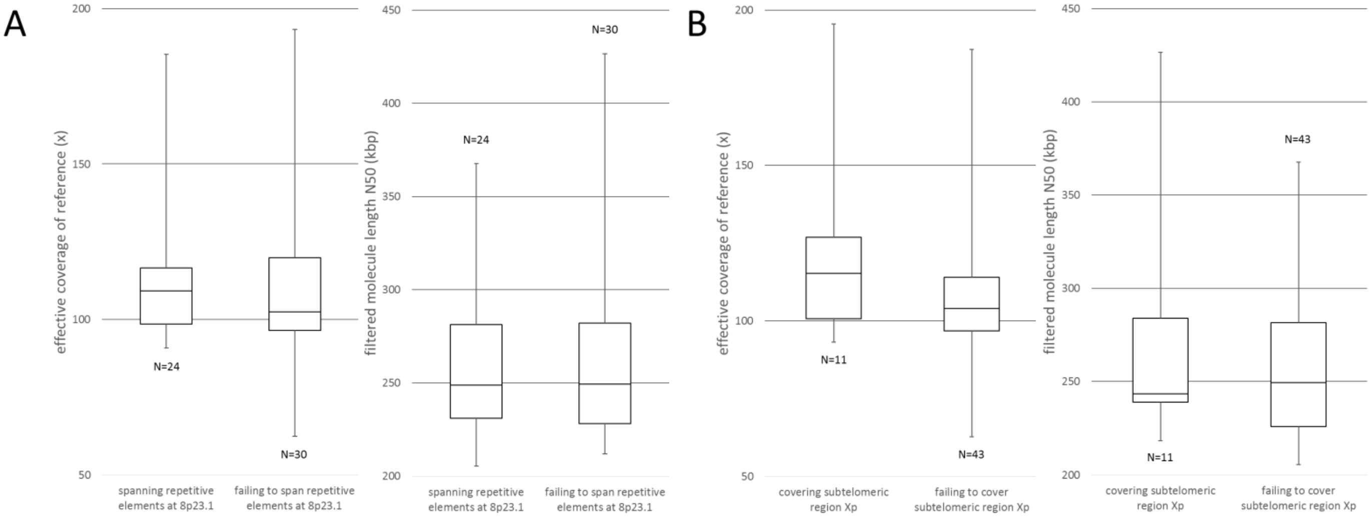

Figure S1: Boxplots illustrating two regions that were difficult to cover. A: Two repetitive elements at 8p23.1 flanking a polymorphic inversion (see Fig 2). At least one repetitive element of at least one allele could be spanned in 24 of the 54 samples included in this study; this was not the case in the remaining 30 samples. The samples are grouped according to the success (left) or failure (right) of spanning the repetitive elements. The comparison between the groups shows that samples with an above-median effective coverage of the reference had a slightly increased likelihood of successfully spanning the repetitive elements. Based on the molecule lengths and coverages we achieved, samples with an above-median molecule length did not appear to have a greater chance of spanning these elements.
B: A portion of the subtelomeric region of Xp (*CSF2R* and the region chrX:352 kbp - 446 kbp). The region covered at least one allele in 11 of the 54 samples included in this study. This was not the case for the remaining 43 samples. The samples are grouped according to the success (left) or failure (right) of coverage of the subtelomeric region. Comparison of the groups shows that the samples with an above-median coverage had an increased likelihood of successfully covering the subtelomeric region. An above-median molecule length could not be shown to have a beneficial effect.
centerline of the boxplot...median; top of the box…3rd quartile; bottom of the box...1st quartile; error bars…lowest and highest value, respectively

Supplementary Methods

Array Comparative Genome Hybridization

The DNA was labeled with the HT Genomic DNA Labeling Kit (Oxford Gene Technology, OGT, Oxford, UK) and hybridized on either a CytoSure Constitutional Array v3 8x60k slide or a CytoSure Medical Research Exome Array 1x1M (OGT) according to the manufacturer's instructions. The slides were scanned using either an InnoScan 710 or an InnoScan 910 AL scanner (Innopsys, Carbonne, France) and processed by the analysis programs Mapix (Innopsys) and CytoSure (Oxford Gene Technology). The data were evaluated with the reference genome GRCh38.

Chromosomal Analysis and Fluorescence In Situ Hybridization

Metaphase preparation from heparin blood samples was carried out by standard methods. Briefly, cells were cultured in LymphoGrow medium (CytoGen, Sinn, Germany) containing phytohemagglutinin as a mitogen. After fixation, metaphases were dropped onto slides and then dried at 60 °C overnight. Metaphase chromosome spreads were evaluated by GTG banding using the karyotyping system Ikaros (MetaSystems, Altlussheim, Germany). For FISH analyses, probes RP11-399J23-orange and RP11-589N15-green from Empire Genomics (Buffalo, NY, USA) and 18pter-orange, SE 18-green, 20pter-orange, SE 13-green, Whole Chromosome 13 and Whole Chromosome 20

from Leica (Wetzlar, Germany) were used according to the manufacturer’s instructions. Images were analyzed using the Isis Digital Imaging System (Metasystem Inc., Altussheim, Germany).

PCR and Nanopore Sequencing

Where applicable, the breakpoints from the OGM analyses were confirmed and further specified by third-generation long-range sequencing using a MinION sequencer (ONT). For this analysis, long-range PCRs were performed for S05 (fwd. primer: 5′-TGCTTGGCCTTTGGGGTATTTTAAATG-3′ and rev. primer: 5′-TGCCACATGTCACAGAGCCAACTC-3′; fwd. primer: 5′-TCCTCACACACATCACTTCTTTACACC-3′ and rev. primer: 5′-TGATGACCATAGCCAAGCTGGTTAAC-3′), S12 (fwd. primer: 5′-GCTGGAGGATCGGTTGTCAACACAG-3′ and rev. primer: 5′-GAGAAGTGCATCAACTCTGACACCA-3′), S13 (fwd. primer: 5′-TCTGAACGCTCCACTGACCAGTCATC-3′ and rev. primer: 5′-ATTCCTACTGTGCCACACTTCTCAGG-3′) and S14 (fwd. primer: 5′-ATATCTTCAGGAGAAGAAGCAGGTG-3′ and rev. primer: 5′-TTCAGGCTCCCTTAAGGAGTATTTTAG-3′). Following PCR amplification, amplicons were prepared for nanopore sequencing on a MinION 106D flow cell according to the manufacturer’s ligation sequencing protocol (SQK-LSK109). Whenever necessary, sequencing results from Nanopore sequencing runs were verified by Sanger sequencing on a Hitachi 3500xL Genetic Analyzer (Thermo Fisher Scientific, Waltham, MA, USA).

MLPA Analysis

Multiplex ligation-dependent probe amplification (MLPA) was performed to verify putative gains and losses when appropriate probe sets were available. For MLPA, the DNA was hybridized with the probes and amplified according to the manufacturer’s instructions. Fragment analysis of amplified DNA was performed on a Hitachi 3500xL Genetic Analyzer (Thermo Fisher), and data were processed with the SeqPilot (JSI, Ettenheim, Germany) analysis program. The MLPA probe sets used for the presented clinical cases were P044-B3 (S12), P034-B2 (S13) and P155-D2 (S14) (MRC-Holland).
